# Treating Status Epilepticus: Phenytoin Versus Levetiracetam

**DOI:** 10.7759/cureus.18515

**Published:** 2021-10-05

**Authors:** Jason Dell'Aquila, Varun Soti

**Affiliations:** 1 Neurology, Lake Erie College of Osteopathic Medicine, Elmira, USA; 2 Pharmacology and Therapeutics, Lake Erie College of Osteopathic Medicine, Elmira, USA

**Keywords:** pharmacokinetics, drug efficacy, status epilepticus, levetiracetam, phenytoin

## Abstract

For decades, phenytoin has been the drug of choice for the treatment of epilepsy but also the second-line treatment for status epilepticus (SE). However, newer antiepileptic drugs (AEDs) have emerged as safer alternatives for the suppression of seizures. Consequently, phenytoin has recently fallen under scrutiny in the research world, prompting many studies to compare its efficacy to these other drugs, most notably levetiracetam. Levetiracetam is a second-generation AED, which is gaining wide clinical use as the second-line agent in treating SE patients. This review focuses on several clinical studies that have directly compared the effectiveness of phenytoin and levetiracetam in suppressing SE seizure activity. Additionally, this review highlights several advantages of using levetiracetam over phenytoin in this clinical context.

## Introduction and background

Epilepsy is a chronic neurological disease that affects approximately 70 million people worldwide [1]. The disease derives from a pathological overexcitation of neurons in the central nervous system (CNS), manifesting acutely as seizures [2]. Despite the lack of a cure, approximately 70% of epilepsy patients can achieve long-term remission with antiepileptic drug (AED) therapy [3]. However, the remaining 30% of patients do not respond to treatment and, therefore, have refractory epilepsy.

Status epilepticus (SE) is the most severe form of an epileptic seizure. It characterizes as continuous and prolonged seizure activity without proper recovery [4]. Between 120,000 and 180,000 episodes of SE occur in the United States annually [5,6]. Representing the most common pediatric neurological emergency, SE occurs more frequently and with a lower mortality rate in children than adults [7]. Additionally, children have lower chances of suffering from neurological sequelae, such as neuronal death, following SE [8]. Conversely, elderly patients tend to be the most severely affected by SE, with a mortality rate approaching 40% [9]. An acute episode of SE is typically treated with various AEDs. Benzodiazepines, such as lorazepam, represent the first line of treatment [10,11]. If seizure activity does not suppress, it is a common clinical practice to prescribe the patient either phenytoin, levetiracetam, or valproate [10-12]. Although phenytoin has traditionally been the drug of choice for the second-line treatment of SE, it has a plethora of side effects, including gingival hyperplasia [13-16]. Consequently, phenytoin usage has declined steadily in recent years despite still being the most frequently prescribed AED [17].

In the hopes of finding a safer alternative to phenytoin, recent studies have compared the efficacy of this AED in treating SE to that of levetiracetam, a relatively newer AED [18-28]. This article provides an overview of the different stages of SE, discusses the use of phenytoin and levetiracetam in treating SE patients, and highlights the key benefits of levetiracetam over phenytoin in treating SE patients.

## Review

**Figure 1:**
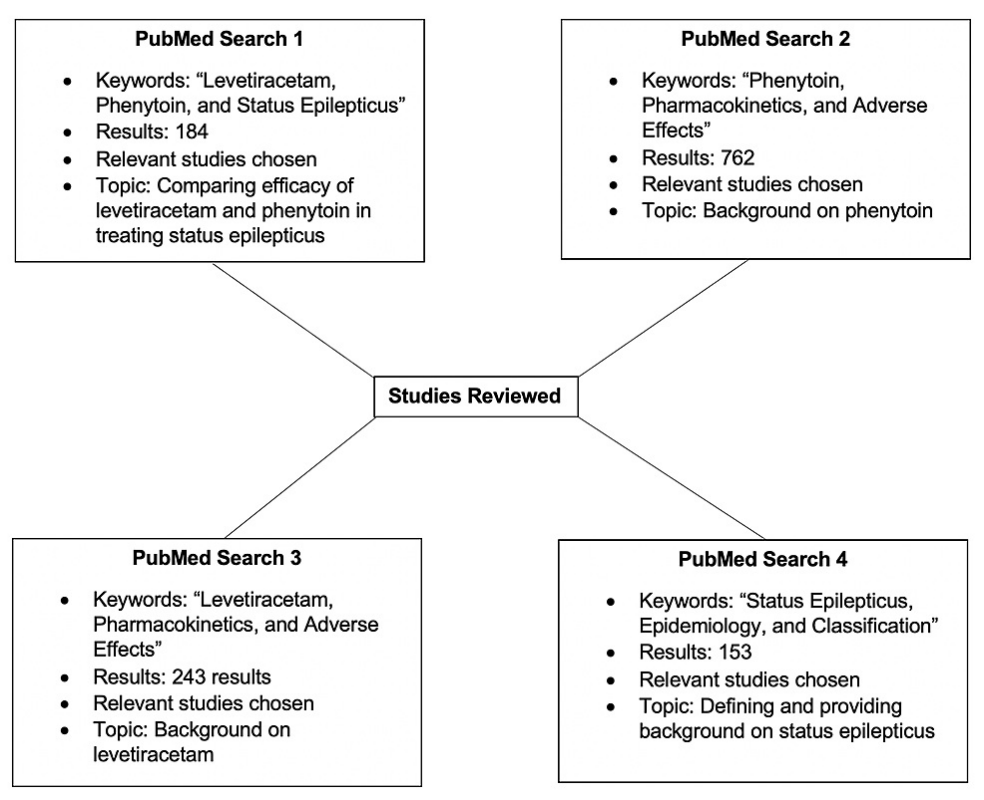
Review methodology Keywords within the same figure box, for example, “Levetiracetam, Phenytoin, and Status Epilepticus” in PubMed Search 1, were entered into PubMed together to increase the specificity and relevance of the results. The studies from PubMed Searches 2, 3, and 4 provided background discussion on the topics, whereas those selected from PubMed Search 1 were the ones primarily reviewed.

We utilized PubMed for the literature search, and articles written only in the English language were selected. The keywords “Levetiracetam, Phenytoin, and Status Epilepticus” yielded 184 results. Furthermore, “Phenytoin, Pharmacokinetics, and Adverse Effects” and “Levetiracetam, Pharmacokinetics, and Adverse Effects” led to the retrieval of 762 and 243 articles, respectively. Lastly, the keywords “Status Epilepticus, Epidemiology, and Classification” generated 153 results. Therefore, we selected relevant preclinical and clinical studies within the scope of this review. Figure 1 illustrates the methodology.

Status epilepticus

The current definition of SE, as stated by the International League Against Epilepsy (ILAE), is “a condition resulting either from the failure of the mechanisms responsible for seizure termination or from the initiation of mechanisms, which lead to abnormally, prolonged seizures (after time point t1)” [29]. The ILAE further defines t2 as the time point after which seizure activity can potentially cause sequelae, including neuronal death, neuronal injury, and alteration of neuronal networks. Research shows that longer seizure duration, cerebral insult, and refractory SE are associated with poor clinical outcomes, implicating the importance of early clinical assessment and treatment [30]. A common catalyst for SE is the discontinuation of AEDs [31]. However, patients who experience SE do not always have a history of epilepsy [7]. In fact, a multitude of conditions can contribute to the onset of SE, including stroke, hypoxia, CNS infection, head trauma, chronic alcohol use, and drug toxicity [31]. There are two primary types of SE: convulsive and non-convulsive [4]. Convulsive SE consists of ongoing convulsive seizure activity without regaining consciousness between seizures for a duration greater than five minutes [32]. Non-convulsive SE lacks convulsions and is typically diagnosed based on abnormal mental status with diminished responsiveness, electroencephalogram (EEG) waveforms characteristic of seizure activity, and responsiveness to AEDs [33].

**Figure 2:**
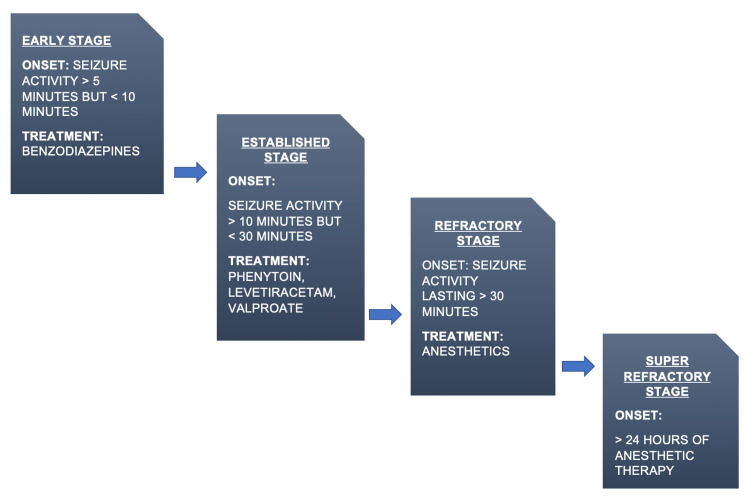
The progression of status epilepticus through four stages Time ranges, as well as treatments for each stage, are highlighted. This review is concerned chiefly with the established stage and its corresponding treatment options.

The clinical progression of SE consists of four stages [34-36]. The early stage of SE begins when continuous seizure activity exceeds five minutes after the treatment with benzodiazepines [4]. Once seizure activity exceeds 10 minutes or is unresponsive to early-stage benzodiazepine treatment, the patient enters the established SE stage. Administer second-line agents such as phenytoin and levetiracetam to treat the patient at this stage [4,10]. SE is refractory when first- and second-line treatments fail, and the seizure activity exceeds 30 minutes [4]. Research has shown that approximately 23% of all SE cases reach the refractory stage [37]. When the seizure activity persists for more than 24 hours or recurs after 24 hours, the patient enters the super-refractory stage [38]. However, very few patients with SE enter the super-refractory stage of SE. Treatment for refractory and super-refractory stages usually consists of anesthetics such as pentobarbital and propofol [39]. Figure 2 illustrates the four stages of SE.

Phenytoin’s traditional use in treating benzodiazepine-resistant SE patients

Phenytoin is currently used and has been in clinical practice for many years to treat patients with SE who do not show any improvement with benzodiazepines [10-12]. Phenytoin, the traditional drug of choice in the management of epilepsy, also helps suppress generalized tonic-clonic and partial-onset seizures [40]. In addition, this AED can serve as seizure prophylaxis after traumatic brain injury [41]. Phenytoin operates on neuronal sodium channels, stabilizing the inactivated state, reducing the sodium influx across the membrane, and curtailing the firing of action potentials and the neuronal overexcitation that leads to a seizure [42]. Phenytoin can be taken orally or administered intravenously (IV) [43]. It is also available as a prodrug, fosphenytoin. It costs more and has a better patient-tolerability profile than regular IV phenytoin [16]. In fact, the most prominent reason why phenytoin is still commonly prescribed is its relatively low cost [44].

Although phenytoin has been an effective AED for decades, its use has gradually declined in recent years, primarily due to its side effect profile [17]. Sedation, hirsutism, gingival hyperplasia, megaloblastic anemia, lupus-like hypersensitivity syndrome, coarsening of facial features, osteomalacia, decreased serum folate, cerebellar syndrome, and locomotor dysfunction are among the significant adverse effects of phenytoin [13]. Moreover, IV phenytoin has additional side effects such as hypotension, cardiac arrhythmia, purple glove syndrome, fatal hemodynamic complications, Stevens-Johnson syndrome, and skin necrosis at the injection site [14-16]. Also, due to phenytoin’s narrow therapeutic index and saturation pharmacokinetics, patients on phenytoin require constant monitoring of their dosing to prevent toxicity [45,46]. Phenytoin metabolizes in the liver by cytochrome P450 (CYP450) enzymes, and it also induces this enzyme system. This action, in turn, increases the clearance of other drugs metabolized by the CYP450 enzymes, leading to interactions with multiple drugs such as warfarin [47,48]. Studies have also shown that genetic polymorphisms in CYP450 enzymes, specifically CYP2C, can increase the serum concentrations of phenytoin and further raise the risk of its adverse effects [49].

Levetiracetam, an emerging alternative to phenytoin in treating SE patients

Having been first introduced to the market in 1999, levetiracetam is a second-generation AED [50]. Common indications for levetiracetam's use are generalized tonic-clonic seizures, partial-onset seizures, and status epilepticus [11,12,51]. Levetiracetam’s mechanism of action is unknown. However, over the years, researchers have proposed several theories describing levetiracetam’s mechanism of action [52-55]. A preclinical study found that levetiracetam removes the zinc-induced suppression of gamma-aminobutyric acid A receptor-mediated presynaptic inhibition, resulting in decreased excitatory transmission [53]. Other preclinical studies have posited a different potential mechanism for this AED, involving the blockade of L-type or N-type calcium channels [54,55]. Despite this uncertainty, research has confirmed that levetiracetam binds to a synaptic vesicle protein called SV2A, leading researchers to postulate that levetiracetam’s antiepileptic activity might derive from the modulation of this protein and its interactions [52,56]. Some preclinical studies have shown that levetiracetam can inhibit epileptogenesis in addition to suppressing seizure activity in ­­­­­­rats. However, there is no such confirmation in human subjects [57].

Unlike phenytoin, levetiracetam does not have many serious adverse effects [15,58]. The most common reported adverse effects are somnolence, asthenia, dizziness, headaches, pyrexia, dry mouth, and behavioral changes [59,60]. In addition, levetiracetam has favorable pharmacokinetics and very few interactions with other medications [61]. Compared to IV phenytoin, IV levetiracetam is easier to administer and has a broader spectrum and lesser side effects [15]. The CYP450 enzyme system does not metabolize levetiracetam, and its bioavailability is close to 100% [50]. Research has shown that levetiracetam has a better patient-tolerability profile even when administered at higher doses and infusion rates [62].

Comparing the efficacies of phenytoin and levetiracetam in treating SE patients

A systematic review of various clinical studies evaluating phenytoin and levetiracetam effectiveness in treating SE patients reveals that levetiracetam is comparable to phenytoin in suppressing seizure activity in SE patients and has fewer adverse effects. Table 1 highlights the findings of clinical studies making efficacy comparisons between phenytoin and levetiracetam in the acute setting to treat SE [18-26].

**Table 1:** Comparison of efficacies of levetiracetam and phenytoin in SE patients The clinical studies meeting the selection criteria were selected, compared, and analyzed. Although phenytoin showed remarkable adverse effects compared to levetiracetam, there was no significant difference between the effectiveness of levetiracetam and phenytoin in treating SE patients. SE, Status epilepticus.

Authors	Type of Study	Title	Sample Size	P-value	Findings
Singh et al. (2018) [18]	Prospective, randomized control	Efficacy of phenytoin versus levetiracetam in suppressing SE seizure activity for 24 hours in children	100 children (3-12 years old)	P = 0.646	There were no significant differences.
Wani et al. (2019) [19]	Prospective, randomized control, nonblinded	Efficacy of phenytoin versus levetiracetam in suppressing SE seizure activity for 24 hours in children	104 children (1 month-12 years old)	P = 0.0001	Levetiracetam was significantly more efficacious than phenytoin.
Chamberlain et al. (2020) [20]	Prospective, randomized control, double-blinded, multicenter, response-adaptive	Efficacies of fosphenytoin, levetiracetam, and valproate in treating SE for different age groups	462 patients: 225 children (<18 years old), 186 adults (18-65 years old), and 51 elderly adults (>65 years old)	P = 0.93	No significant differences between phenytoin and levetiracetam groups across age groups.
Mundlamuri et al. (2015) [21]	Prospective, randomized control	Efficacies of levetiracetam, phenytoin, and valproate in treating SE	150 patients	P = 0.44	There were no significant differences.
Appleton et al. (2020) [22]	Prospective, randomized control, open-label	Efficacies of levetiracetam and phenytoin in treating established convulsive SE	286 children (6 months-17 years old and 11 months old)	P = 0.2	There were no significant differences.
Noureen et al. (2019) [23]	Prospective, randomized control, open-label	Efficacies of levetiracetam and phenytoin in suppressing SE seizure activity for 30 minutes	600 children	P = 0.0128	Levetiracetam was significantly more efficacious than phenytoin.
Chakravarthi et al. (2015) [24]	Prospective, randomized control	Efficacies of levetiracetam and phenytoin in suppressing SE seizure activity for 30 minutes	44 adults	P = 0.53	There were no significant differences.
Besli et al. (2020) [25]	Retrospective, non-randomized, nonblinded	Efficacies of levetiracetam and phenytoin in treating convulsive SE and acute repetitive seizures in children	277 children (1 month-18 years old)	P = 0.011; P = 0.791	Convulsive SE: Levetiracetam was significantly more efficacious than phenytoin. Acute repetitive seizures: There were no significant differences.
Nakamura et al. (2017) [26]	Retrospective, non-randomized, nonblinded	Efficacies of levetiracetam and fosphenytoin in preventing recurrence of seizures after the termination of SE	63 adults	P = 0.69	There were no significant differences.

Singh et al. measured the efficacies of both phenytoin and levetiracetam in suppressing seizure activity for 24 hours in 100 children presenting with acute seizures in a randomized control study. Patients between the ages of three and 12 years were randomly assigned to phenytoin and levetiracetam groups, with each group consisting of 50 patients. Children convulsing upon admission to the emergency room received IV diazepam, a benzodiazepine, before further treatment in each group. Although there were no significant differences in efficacy between the two drugs, levetiracetam achieved 100% therapeutic levels after one hour and 98% after 24 hours compared to 76% therapeutic levels achieved by phenytoin at four and 24 hours [18]. However, an accurate comparison between the two groups cannot be made because the two drugs were measured at different time intervals, levetiracetam after one hour and phenytoin after four hours. In addition, more patients in the phenytoin group had lower diastolic blood pressure.

Wani et al. also compared the efficacies of phenytoin and levetiracetam in treating children presenting with SE. This study was prospective, randomized controlled, and nonblinded and was carried out with 104 children between one month and 12 years, with the seizure control measured over 24 hours. Interestingly, levetiracetam significantly suppressed seizures compared to phenytoin [19]. However, the sample size was relatively small, reducing the statistical power of the findings. In addition, the study was nonblinded, allowing for potential bias in the assessment of the patients.

In a recent study, Chamberlain et al. assessed the efficacies of levetiracetam, fosphenytoin, and valproate for treating SE in patients of different age groups [20]. It was a double-blinded, multicenter, response-adaptive study with a randomized controlled design, enrolling 462 people: 225 children (18 years and younger), 186 adults (aged 18-65 years), and 51 elderly adults (over the age of 65). This study randomly assigned patients to each treatment group: 175 patients to the levetiracetam group, 142 patients to the fosphenytoin group, and 145 to the valproate group. All patients had established SE. Consistent with previous findings of Wani et al., which was a smaller study, this study showed no significant differences in fosphenytoin, levetiracetam, and valproate efficacies across the age groups [19]. Also, there was no remarkable difference between fosphenytoin and levetiracetam efficacies in the study subjects [20]. This study had a robust design as well as a large sample size.

A study by Mundlamuri et al. also investigated the differences in the efficacies of levetiracetam, phenytoin, and valproate in treating SE patients. This randomized controlled trial enrolled 150 patients, with 50 patients assigned to each treatment group. All patients received lorazepam before receiving second-line treatment. The researchers did not control for patient age. This study also showed no significant differences in the efficacy of the three drugs in treating benzodiazepine-resistant SE patients [21]. However, this study had a relatively small sample size and did not exclusively compare levetiracetam and phenytoin.

Appleton et al. conducted a randomized controlled trial comparing the efficacy of levetiracetam and phenytoin in children with established convulsive SE. The study enrolled 286 children between six months to 17 years and 11 months, with 152 children receiving levetiracetam and 134 receiving phenytoin. Interestingly, 70% of children in the levetiracetam group showed decreased seizure activity than 64% in the phenytoin group. However, this difference was not statistically significant [22]. In addition, this study introduced potential bias from a design standpoint by employing an open-label rather than a double-blinded paradigm.

In another open-label, randomized controlled trial, Noureen et al. enrolled and randomized 600 children with established SE into levetiracetam and phenytoin groups. The primary outcome was the cessation of seizure activity within 30 minutes of drug administration, and the secondary outcome was the presence of adverse effects. The study findings showed levetiracetam was more effective than phenytoin in suppressing seizures. However, eight patients experienced cardiac and respiratory depression after being treated with phenytoin compared to those who received levetiracetam [23]. Although the study revealed the difference between the number of patients experiencing adverse effects between the two groups, it had obvious flaws. First, instead of utilizing a double-blind design, it employed an open-label model. Second, it only assessed the suppression of seizure activity 30 minutes after drug administration. Furthermore, the researchers overlooked the potential of seizure recurrences.

Chakravarthi et al. conducted a randomized controlled trial in which levetiracetam and phenytoin showed similar effectiveness in treating SE. The primary outcome measure was successful termination of seizure activity 30 minutes after drug administration. Secondary outcome measures included recurrence of seizures after 24 hours, mortality during hospitalization, need for ventilatory assistance, and neurological state at discharge [24]. However, the sample size was small as the trial enrolled only 44 patients. Additionally, this study did not control for patient age, further confounding the results.

In a retrospective study, Besli et al. compared the efficacy and safety profile of levetiracetam and phenytoin to treat convulsive SE and acute repetitive seizures in 277 children between one month and 18 years. Unlike those with SE, patients with acute repetitive seizures regain consciousness between seizures. While there was no disparity between levetiracetam and phenytoin in treating acute repetitive seizures, levetiracetam significantly showed more seizure suppression activity in SE than phenytoin. Additionally, phenytoin caused adverse reactions, notably hypotension. There were no such adverse reactions with levetiracetam [25]. However, this study had limitations due to its retrospective design.

Nakamura et al. also juxtaposed the efficacy of levetiracetam and phenytoin. However, rather than focusing on the suppression of SE seizure activity in the acute setting, this study looked at the effectiveness of each drug in preventing recurring seizures after diazepam successfully terminated SE. The researchers looked at the medical records of 63 patients, with 21 and 42 receiving levetiracetam and fosphenytoin, respectively. The mean patient age was 64 years. Both drugs similarly precluded seizure recurrence with no significant difference. This study also focused on the presence of adverse effects and the ease of transition from IV to oral routes for both drugs. Reduction of blood pressure was observed in response to fosphenytoin but not levetiracetam, and the transition of treatment method was more efficient in the levetiracetam group [26]. Although this study was retrospective and had a small sample size, its findings corroborated previous studies showing no significant difference in efficacy between levetiracetam and phenytoin.

When taken together, these results suggest that levetiracetam is very similar to phenytoin at efficaciously treating SE. Interestingly, Wani et al., Noureen et al., and Besli et al. showed that levetiracetam has greater efficacy than phenytoin [19,23,25]. However, a recent meta-analysis found no significant difference in efficacy between the two drugs in treating status epilepticus [27]. Given the high statistical power of this study and the fact that most clinical studies corroborate these findings, it is likely that levetiracetam and phenytoin are very similar in their ability to suppress seizure activity in SE effectively.

Benefits of using levetiracetam over phenytoin

Despite the similar efficacies of the two drugs in treating SE, numerous studies have demonstrated that levetiracetam is a safer AED to administer than phenytoin [15,18,23,25,26,63]. Four of the studies in Table 1 reported a significantly greater incidence of adverse effects associated with phenytoin treatment than levetiracetam, with the most common being acute hypotension [18,23,25,26]. Another clinical study showed that IV fosphenytoin was associated with significantly greater vasopressor usage than levetiracetam when treating SE, primarily due to the hypotension induced [63]. Since maintaining cerebral blood perfusion is vital in helping to prevent neuronal injury in SE, the risk of hypotension associated with IV phenytoin and fosphenytoin could lead to worse patient outcomes [64]. Additionally, clinical studies show that IV phenytoin can cause potentially fatal cardiac arrhythmias [14-16]. Although initially thought to reduce the risk of cardiac toxicity, fosphenytoin also appears to induce cardiac arrhythmias [65]. Conversely, levetiracetam has a less severe adverse effect profile and appears to be well tolerated in diverse populations for the treatment of SE [15,58-60,62].

Other advantages of using levetiracetam over phenytoin to treat SE are its more favorable pharmacokinetics and relative ease of administration [15,50,61,66]. Unlike phenytoin, levetiracetam is not metabolized by the CYP450 enzyme system in the liver, substantially reducing chances of levetiracetam interactions with other drugs metabolized by CYP450 [47,48,50]. In addition, levetiracetam exhibits a bioavailability of nearly 100% and, in contrast to phenytoin, does not require constant monitoring of dosing [50]. Another advantage of levetiracetam is its linear pharmacokinetics and broad therapeutic index, dramatically reducing the risk for drug toxicity [66]. Importantly, levetiracetam is relatively easier and faster to administer than phenytoin [15,67]. In fact, giving a loading dose of IV phenytoin to a patient is a lengthy and time-consuming procedure, further increasing the risk for adverse effects [67]. The efficacies of the two drugs in treating SE may be similar, but levetiracetam is the safer and better tolerated AED. Therefore, it should replace phenytoin and fosphenytoin for terminating seizure activity in benzodiazepine-resistant SE patients.

## Conclusions

Over the years, phenytoin has been successful in managing SE patients. However, newer AEDs, including levetiracetam, have emerged as alternatives. Researchers have mostly tried to compare and analyze the efficacies of the two drugs in treating SE patients and have not focused on implementing relative safety measures. This review sheds light on several of these studies and variabilities across clinical trials in patient outcomes and trial designs. There were no significant differences between phenytoin and levetiracetam in their efficacies in treating SE patients. However, levetiracetam showed a substantially lower incidence of adverse effects compared to phenytoin. Moreover, levetiracetam offers numerous advantages over phenytoin, such as less dose monitoring, fewer drug interactions, achieving therapeutic levels faster, and a broader therapeutic index. With these benefits, levetiracetam is slowly gaining wide clinical use, in some instances, replacing phenytoin. Nevertheless, more research is required to further elucidate levetiracetam's relative efficacy in treating SE patients, particularly adults. Improving patient outcomes is essential when selecting a treatment, and using levetiracetam as an alternative to phenytoin for SE may prove to be a massive step.
